# Acculturation, Health Behaviors, and Social Relations among Chinese Immigrants Living in Spain

**DOI:** 10.3390/ijerph18147639

**Published:** 2021-07-18

**Authors:** Barbara Badanta, Juan Vega-Escaño, Sergio Barrientos-Trigo, Lorena Tarriño-Concejero, María Ángeles García-Carpintero Muñoz, María González-Cano-Caballero, Antonio Barbero-Radío, Domingo de-Pedro-Jimenez, Giancarlo Lucchetti, Rocío de Diego-Cordero

**Affiliations:** 1 Research Group PAIDI-CTS 1050 Complex Care, Chronicity and Health Outcomes, Faculty of Nursing, Physiotherapy and Podiatry, University of Seville, 41009 Seville, Spain; bbadanta@us.es (B.B.); ltarrino@us.es (L.T.-C.); agcarpin@us.es (M.Á.G.-C.M.); 2 Research Group PAIDI-CTS 1054 Interventions and Health Care, Red Cross, Spanish Red Cross Nursing School, University of Seville, 41009 Sevilla, Spain; juanvegadue@gmail.com; 3 Research Group PAIDI-CTS 969 Innovation in HealthCare and Social Determinants of Health, Faculty of Nursing, Physiotherapy and Podiatry, University of Seville, 41009 Seville, Spain; abarbero1@us.es (A.B.-R.); rdediego2@us.es (R.d.D.-C.); 4 University of Cádiz, 11003 Cádiz, Spain; dodepeji@gmail.com; 5 School of Medicine, Universidade Federal de Juiz de Fora, Juiz de Fora 36036-900, Brazil; g.lucchetti@yahoo.com.br

**Keywords:** acculturation, emigration and immigration, health behaviors, qualitative method, Spain

## Abstract

This study aims to identify acculturation experiences about social relations and health behaviors of first-generation Chinese immigrants in the South of Spain, including food patterns, physical exercise, and tobacco and alcohol use. A phenomenological qualitative study was conducted using semi-structured interviews, informal conversations, and field notes. All data were analyzed under the Berry’s Model of Acculturation. A total of 133 Chinese immigrants were included. Our findings show that separation was the dominant acculturation strategy, followed by integration and assimilation, while marginalization was not present in this immigrant population. Most of the immigrant population maintains a link to the customs of their home country, favoring the process of identity and collective self-esteem. These results can help health managers and the government to further understand Chinese immigrants in Europe and to establish appropriate health interventions to this group.

## 1. Introduction

According to the Migration Data Portal, the number of international migrants in 2019 reached 271.6 million worldwide as compared to 258 million in 2017. In the European Union, Spain ranked fourth (7%) in the reception of immigrants [1]. The immigration process can be associated with two different conditions. The first one is based on the forced immigration (i.e., asylum seekers or refugees), which may result in mental health problems and infectious diseases to immigrants [2]. The second one is the non-forced immigration (i.e., those living and working in the host country), which tends to promote better life conditions, resulting in challenges towards the degree of integration into the host communities and other social aspects such as work activity or territorial concentration. The latter is considered the case of the Chinese immigrant community in Spain [3,4].

In fact, the entry of Spain into the European Economic Community in 1986 made this country a good destination for the expansion of businesses by the Chinese population [5]. Spain has now occupied the fourth place in the European ranking in terms of immigrants from China [6]. From 2005 to the present date, Chinese individuals represent the most common immigrants from Asia [7], constituting the fifth largest nationality of immigrants in Spain (4.46%) [8].

Since their arrival in Spain, this population has established several Chinese restaurants, which promoted the migration of this population to the most important cities of the country and the coastal area. In an attempt to guarantee a strategic location, minimize the competition, and search for new market opportunities, first-generation Chinese immigrants began the so-called “first inward expansion” in the 1990s. Therefore, they migrated to other large cities, which explains the presence of Chinese population in Madrid, Catalonia, and the Canary Islands in the 1980s, followed by the Valencian Community and Andalusia five years later [9,10].

In 2019, the Chinese population living in Andalusia constituted 9% of the total Chinese immigrants in Spain, being the third largest foreign nationality (22,280 inhabitants), behind Moroccan (145,076 inhabitants) and Romanian (79,264 inhabitants) immigrants. Likewise, although Spain is experiencing a decline in the immigrant population of other nationalities due to the economic crisis that Spain suffered since 2008 [6], the Chinese presence in Spain has maintained a steady growth.

According to the “Melting Pot” adaptive theory [11], upon arrival in the host country, immigrants gradually take on cultural aspects and build a new “cultural form”, which may have an important influence on their individual, social, and contextual factors. Immigrants usually change the way they dress, what they eat, their greeting habits, and even their values by reducing (i.e., suppressing, forgetting) their way of living. The pace and extent of individual change is related to the degree of cultural maintenance in one’s own group, which in turn is linked to the relative demographic, economic, and political situation of the host community [12].

This difficult process called acculturation is influenced by the culture of origin (i.e., religion, language, education), the policies of the hosting country’s government [13], age, socioeconomic status, and even by offspring [14]. Although it involves at least two groups, with consequences for both, there is a greater impact for the nondominant group [12].

Social relationships, including the use of language, as well as the acquisition of certain lifestyles, have been used to measure acculturation in Asiatic populations [15], and considering the role of acculturation on immigrant social relations [15] and health behaviors [16,17] is capital for the healthcare of the immigrant population. 

Acculturation has been extensively addressed by previous studies. In an Australian study, male immigrants from North Africa/Middle East and Oceania regions were susceptible to weight gain, with higher levels of acculturation being negatively associated with being overweight [18]. In another study, Schotte et al. investigated the association of cultural identity with several indicators of academic achievement and psychological adaptation among immigrant adolescents in Germany, showing that the identification with both the mainstream context and the ethnic context were important factors related to a positive development and adaptation of immigrant adolescents [19].

Concerning the Asiatic immigrant population, the relationship between the acculturation effect and the health lifestyles has been already investigated in the U.S.A, showing that the health risks to this group of immigrants increased due to the North American lifestyle, which, in other words, reflects the negative effect of acculturation. Nevertheless, the mechanisms underlying this relationship are still understudied [20]. In a study with Chinese international students in the Midwest of the U.S.A, the authors found that the stress due to acculturation is a risk factor for substance use. However, on the other hand, bicultural affiliation reduces the likelihood of smoking, drinking, and getting drunk [21].

Despite the previous evidence, there are few studies that address the phenomenon of acculturation related to health. In a recent systematic review, acculturation was associated with mental health aspects such as anxiety, disruptive behaviors, psychological adaptation, satisfaction with life, and emotional exhaustion [22]. Nevertheless, other health variables are seldom investigated in this field of research.

In this context, a recent European concern is the high number of Asian immigrants settled in Spain, particularly Chinese immigrants [23]. However, there are still few studies that have assessed how the migration phenomenon itself may affect the health of these individuals and, to our knowledge, this is the first study that addresses this aspect among first-generation Chinese immigrants in Spain.

This study provides further knowledge on the acculturation strategies that are beneficial or detrimental to the health of immigrants. This will allow health managers and healthcare professionals to design and promote healthcare interventions and to prevent unhealthy lifestyle behaviors among immigrants. In addition, healthcare services should be configured taking into account the essential cultural variables of the Chinese immigrants, since these variables could be determinants for the success or failure of healthcare in this population.

### Theoretical Framework 

Several theories have studied the immigrant integration into host societies using theories and concepts such as immigrant acculturation and adaptation [24]. This study considers acculturation as “a process of group and individual changes in culture and behavior that result from intercultural contact” [25].

The acculturation model used by this study was first proposed by Berry [24] and is considered a bidimensional model, which is based on the fact that the acculturation entails two independent dimensions: maintenance of the culture of origin and adherence to the dominant or host culture. This theoretical perspective proposes that immigrants can adopt up to four possible adaptation strategies: (a) assimilation, when the immigrant abandons his identity of origin and acquires that of the majority group; (b) integration or biculturalism, when there is a strong identification with both societies or cultures, so that the immigrant preserves the characteristics of their culture, but also participates or shares the culture of the majority group; (c) separation, when the immigrant does not try to establish relationships with the majority group and seeks to reinforce their ethnic identity and; (d) marginalization, in which the immigrant loses his native cultural identity, and also does not want or does not have the right to participate in the culture of the dominant group [12,26]. These aforementioned strategies are based on the idea that immigrant groups and their individual members have the freedom to choose how they want to engage in intercultural relations [12].

Therefore, this study aims to identify unique acculturation experiences and describe their influences on social relations and health behaviors among first-generation Chinese immigrants (foreign-born population who emigrated to Spain when they were children, adolescents, or adults) in the South of Spain, including food patterns, physical exercise, and tobacco and alcohol use.

## 2. Materials and Methods

### 2.1. Design

A qualitative, exploratory, and descriptive design using a phenomenological approach [27] was conducted in the southern region of Spain. This design allowed us to explore a particular topic of interest in a specific context, and to perform an analysis focusing on subcultural groups rather than involving entire societies.

In the present study, we opted to use the Berry’s Model of Acculturation as described above [26]. Despite several theories and models of acculturation used for research, bidimensional approaches such as Berry’s model may better conceptualize acculturation and explain in more detail the health habits as compared to other unidimensional approaches [28].

Data collection consisted of semi-structured interviews with Chinese immigrants, informal conversations, and field notes, and all were carried out by the main researcher (B.B) over six months in 2016/2017.

### 2.2. Data Collection

Our study took place in Andalusia, the southernmost region of Spain and Europe. The focus was on participants’ shared behaviors and experiences; thus, we worked under the assumption that they share cultural perspectives, even if they do not know each other. Participants were recruited through Chinese businesses (e.g., bazaars, restaurants, grocery stores, fashion stores, technology stores, and wholesale businesses) and community institutions (e.g., educational institutions, Asian cultural centers, and health services). In order to increase the number of participants, a “snowball sampling” procedure was also used. This is a valid method to conduct face-to-face interviews while investigating an ethnic minority population [29]. Participants were included in the study if they were adult immigrants of Chinese origin, emigrated to Spain, and were able to communicate in Mandarin Chinese, Spanish, or English.

Semi-structured interviews were carried out face-to-face and lasted between 30 to 60 min. Statements of informed consent for all participants were obtained. The goal of the semi-structured interviews was to create the framework for the participants in which they were comfortable to talk about sensitive issues, while also giving the researcher the opportunity to ask for elaborations about specific topics, explanations of observed events, and clarification of ambiguities. All the interviews were audiotaped and transcribed verbatim by the main researcher (B.B) and data collection continued until criteria saturation.

Information from field notes and informal conversations were also included concerning witnessed events, verbatim verbal exchanges, and the researcher’s personal interpretations of events. Informal conversations and the interviews allowed the researcher to examine whether interpretations of meanings behind observed behavior coincided with participants’ own understandings. All interviews were conducted using the following starting open questions: “What were the reasons for the migration? What has been your experience during migration and upon arrival in the host country? And over the years? What kind of relationships do you have with the Spanish population and your ethnic group? Since you are in Spain, what eating habits do you have? And what about physical activity? Have you started or changed alcohol or tobacco use?” All the questions were agreed upon and discussed among the authors, taking into account the theoretical aspects that explain acculturation and acculturative strategies (Table 1). A consensus among researchers was reached on these open questions. After this first stage and before interviewing the participants, two native Chinese professors who speak Spanish provided feedback on the interview script. Grammatical errors were detected and corrected, and this version was considered appropriate and understandable for the Chinese population. When the first two interviews were transcribed, they also verified the adequacy of the answers to the questions, determining the reliability of the script.

### 2.3. Data Analysis 

The qualitative analysis was carried out following the steps proposed by Braun et al. [30]: (1) familiarization with the data; (2) generation of categories; (3–5) search, review, and definition of themes; and (6) the final report. The data obtained were captured through audio recording and with the use of a field diary. Since some statements were recorded in Chinese, the following translation process was carried out: a Chinese-English translation by a Chinese native (*n* = 2) and an English-Chinese back-translation by a translation company.

Transcription, literal reading, and theoretical categorization were performed, and the NUDIST Nvivo (version 12) software (QSR internacional, Melbourne, Australia) was used. Data analysis started with individual readings in order to get an overview of respondents’ experiences. Two researchers read all field notes and interview transcriptions several times, to gain an overall understanding of the content. The other authors read samples of the field notes and interviews to obtain understanding. The analysis continued by organizing descriptive labels, focusing on emerging or persistent concepts and similarities/differences in participants’ behaviors and statements. The coded data from each participant were examined and compared with the data from all the other participants in order to develop categories of meanings.

When a basically clear pattern emerged with respect to the ideal types of acculturation strategies [26], it was used to critically reflect the data and theoretically frame the results. Furthermore, numerous contextual factors influence the trajectory of their adaptation to a new society. To overcome this limitation, social patterns and contextual factors were carefully considered in the measurement process [31] (see Supplementary Materials—Table S1). Two main themes *(“Social Relations”* and *“Health Lifestyles”*) reflected all of the assessed domains. A final report was prepared with the statements of the Chinese immigrants displayed in the following format: “C-questionnaire number, sex, age”.

This research followed the criteria of The Consolidated Criteria for Reporting Qualitative Studies (COREQ) (Supplementary Materials—Table S2). The methods used in order to guarantee quality were data triangulation, including participants with different sociodemographic characteristics, and triangulation of data analysis via different researchers.

### 2.4. Ethical Considerations

The study was approved by the Andalusian Research Ethics Committee, Spain (Code: 0873-N-16). All participants received written and oral information about the study, including the right to withdraw and the guarantee of anonymity. Data were anonymized by removing names/locations and by changing details. Interview transcripts and audiotapes were kept in locked files.

## 3. Results

Based on the migratory experiences of the participants, the results present a contextual, detailed, and graphic description of the general characteristics of the migratory process to Spain experienced by the Chinese immigrant population. In this section, we first present a sociodemographic analysis of the participants, and second we present the perspective of Berry’s Model of Acculturation and its interface with health, including two themes and five domains retrieved by the qualitative analysis.

### 3.1. “We Want to Earn Money to Improve the Living Conditions of Our Family”: The Migratory Adventure

A total of 252 businesses and institutions were visited and 133 Chinese immigrants agreed to participate. The sample included only Chinese immigrants and consisted of 61.7% women and 38.3% men, with a mean age of 30.7 years (ranging from 18 years to 55 years old), and an average length of residence in Spain of 11.3 years. More details about the participants are shown in Table 2.

The sociodemographic characteristics of the participants reveal that most of them come from the Zhejiang province. Zhejiang is a rural geographical area, with few job and economic opportunities for its inhabitants, especially for those with a lower educational level. This is the main reason why Chinese families with low qualifications among their members decide to emigrate. Given that migration for economic reasons is unanimous, immigrants tend to share this decision with other family members. The immigration usually works in the following way: one of the marriage partners emigrates alone and provides economic support to their family in China, waiting until the whole family (including children) is able to emigrate to Spain. Most immigrants use airport transportation to travel to Spain, which does not initially imply risks to their health.

The migration experience of their peers allows Chinese immigrants to search within their community for a support network that makes migration and adaptation to the destination a more comfortable and easier process. Social relations between Chinese people allows for offering work within the same ethnic niche, the maintenance of the original language, and sharing the same culinary customs, among others.

**Figure 1:**
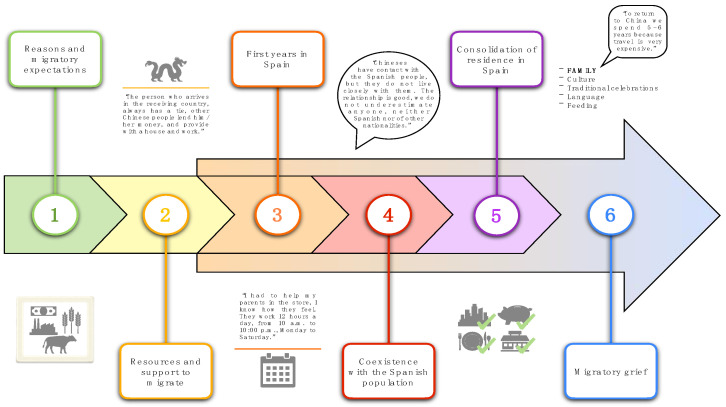
Milestones associated with the Chinese migratory process.

From the statements of the participants, the most important milestones associated with the migratory process are shown in Figure 1.

### 3.2. Berry’s Model of Acculturation and Health

As a result of the acculturation process, social and health behaviors were analyzed under the perspective of Berry’s Model. In the present study, two main themes (“*Social Relations”* and *“Health Lifestyles”*) and five domains (“*Values and need: Social relations among Chinese”, “Experiences within Spanish society”, “Food pattern”, Physical Activity”,* and *“Tobacco and alcohol”*) were retrieved and are described below.

#### 3.2.1. Theme: Social Relations

1. Domain: Values and need: Social relations among Chinese

In our analysis, cultural separation is the most used strategy for social relationships among Chinese immigrant community members, since they respond affirmatively to maintaining strong ties with the group of origin, while contact with people of the new culture is still scarce. This seems to be partially explained by one of the fundamental pillars of the Confucian Chinese philosophy, which is the value given to the family. To these participants, Chinese society is understood as a “large family”, with hierarchical standards of respect and courtesy towards important family members.

*“There is the concept of guānxi, so reputation is very important (...). A Chinese who only knows another Chinese twice and invites him to the wedding… he is almost obliged to go, because if he refuses, the community will judge this act inappropriate and consequences may arise”* (C-48 man, 40 years).

In addition to these values, the degree and quantity of social relations among Chinese individuals is not an option, but rather an imperative aiming to guarantee support and resources within the community and, for this reason, assimilation or marginalization strategies seem to have no place:

*“The range of social relations that a Chinese has is very large (…) the more friends you have at all levels of society, the better, because Chinese society works like this”* (C-78 woman, 22 years).

Likewise, Chinese immigrants usually need support from other Chinese immigrants in order to be able to settle in the destination cities upon arrival in Spain:

*“They offer housing, food and they help other Chinese to find a job (...). It is not enough when you want to start your own business, so your cousins, your brothers, everyone helps you”* (C-21 man, 30 years)*; “The support is fundamentally financial and it comes from the family, friends and acquaintances, they do not usually request external financing. Furthermore, this is done without any claim to profit (...). It is a society in which commitment and loyalty are highly taken into account and everyone knows that favors will be returned”* (C-94 woman, 41 years).

Having secured financing is very important for them in the decision to emigrate and also to work for others and then open their own business, which is their ultimate goal. This reflects that relations with the ethnic group are not temporary, but are maintained over time, since those who one day received help will help others in the future.

2. Domain: Experiences within Spanish society

Chinese immigrants perceive prejudices from Spanish population:

*“People [Spanish people] say that we are invading, we are stealing their jobs, we do not pay taxes; that’s a lie!”* (C-120 man, 19 years).

The lack of cultural integration with the Spanish population seems to have deleterious effects to the Chinese community, which has difficulties in understanding the host culture and tends to reject contact with those who judge them in this negative way.

It seems that community support alleviates the psychological discomfort of being exposed to constant prejudice. In this respect, most participants reported that they were satisfied with their current life in Spain, and highlighted positive aspects such as the emotional support received by their peers, having people who care about them, receiving love and affection, being able to talk about their problems, receiving useful advice, and receiving invitations to go out.

Anther barrier for social relations with the Spanish population is the language, as noted below:

*“If you do not know the language, you are secluded (...), and if you are working in an environment in which you use the language minimally, you do not learn it”* (C-35 woman, 19 years)*; “They have a hard time learning Spanish. I have been Spanish teacher for adult Chinese for more than 10 years, but there is no way they can learn it well”* (C-127 woman, 44 years).

*“I always tell parents that they should bring their children before, when they are little. It is very difficult to learn a new and so different language when the child is 12 years old (…). I think that when they are small, they are abandoned in China, and when they grow up, they are abandoned by society”* (C-72 woman, 43 years).

Finally, work seems to be a key factor in initiating ties with the Spanish population, since relationships are “forced” because Chinese immigrants need to attend to customers in their businesses. In the case of younger Chinese individuals, this relationships tend to be stronger due to their school friends.

*“I believe that Chinese immigrants make relationships, especially among the Chinese members, united by the labor issue and with their family. Although, they can also make any relationship with a Spanish person through work, or a family member, there are people who do not have good experience and feel indifferent and protect themselves in their own community. But then, there are other people and there are more and more young Chinese who have been born here, who go to schools. In short, in 15–20 years this will change a lot”* (C-40 man, 53 years).

#### 3.2.2. Theme: Health Lifestyles

1. Domain: Food patterns

There is a clear barrier concerning Chinese immigrants to abandon their culinary habits of origin or to incorporate at least in part the Spanish cuisine.

*“The food is totally Chinese from the beginning of the day. We usually have boiled rice or noodle soup for breakfast”* (C-88 man, 35 years).

Their basic diet is characterized by the use of many dishes with varied products, the daily consumption of rice or noodles as the main source of carbohydrates, the consumption of vegetables, as well as the frequent consumption of meat, fish, and eggs, with different ways of cooking (wok or dehydration) as compared to the Western tradition. All these products are available in Spanish supermarkets and in other specialized markets owned by the Chinese community.

Food cultural separation is clearly supported when healthier characteristics are attributed to the Chinese diet compared to the Western one, such as the regulatory capacity of foods to ensure the balance between cold and heat and the “yin and yang” associated with Traditional Chinese Medicine (TCM). Another factor related to the maintenance of these customs is the presence of many typical dishes associated with festivals of Chinese culture and the attribution of properties such as luck.

Something observed during the study was that as the time living in Spain increases, Chinese immigrants tend to further integrate Western foods (mainly toast, cereals, or coffee with milk) into their meals such as breakfast. There are a greater number of Chinese younger individuals eating the Western breakfast, since this is more practical and fast in their view:

*“I usually have Spanish breakfast, milk with cereals, since I work very early and I don’t have time to prepare an authentic Chinese breakfast. To do this it would need at least an hour [to cook the rice soup and eat it]”* (C-63 woman, 22 years).

Western fast food and fat consumption is also more prevalent among young people and is even provided to young children by their parents in business. These signs of cultural integration are also manifested in the adherence to the Western body worship and beauty, which affects diets carried out by women and young people, the former restricting certain foods to lose weight, and the latter incorporating hyperprotein diets that support the physical activity performed in gyms.

Despite the fact that Chinese immigrants are incorporating some Western foods, all participants reported that they still prefer their original Chinese food:

*“We can go to a Spanish restaurant one day and eat tapas, but not daily”* (C-36 man, 37 years).

2. Domain: Physical activity

Although the practice of regular exercise in parks and squares in China is common, this is not maintained by Chinese immigrants living in Spain. Likewise, the choice of exercise by participants is minimally influenced by the traditional Chinese culture and, for this reason, the group practice of GuangBo TiCao (group exercise characterized by collectivity, discipline and conformity), tai chi, Yuanji dance, and kung fu are not very common in Spain. The scarce physical activity carried out by this group also does not maintain many similarities with the practice of the Spanish population, and is mainly related to the lack of time due to the intensity of their working day:

*“Sometimes I ran 3 or 4 times a week at night, but now between the store and the girl, I can’t do it much”* (C-43 woman, 36 years).

The Chinese immigrants’ concerns on their job and the work overload decrease the importance of physical activity as a source of physical well-being. In addition, Chinese consider themselves to be physically active during their working day, reporting that they practice a lot of exercise during the tasks they perform in their jobs.

*“Intense physical activity is what I do for 3 h, once a week when I unload the truck with the merchandise. In addition, all week I have moderate activity when I have to go shopping at the stores to replenish the daily basic merchandise”* (C-5 man, 43 years).

As a result of acculturation, young people are again those who show a greater integration of habits related to physical activity and sport. It is common for young people to use the gyms and perform more intense physical activities such as weight-lifting.

*“I think most of the young people go to gyms and play sports, but most adults do not practice any physical activity (...). If they leave in the morning and return at night and also have family responsibilities ... no”* (C-44 man, 26 years).

3. Domain: Tobacco and alcohol

Chinese immigrants consider smoking as a social behavior, particularly for men: (KI-5) “*The idea of them is that to do business you have to smoke and drink*”, and they believe Chinese individuals have a greater consumption compared to the Western population. Although most smokers maintain a consumption of more than one pack of cigarettes a day, they are forced to obey certain Spanish legal regulations, such as the prohibition of smoking inside establishments. Nevertheless, some establishments such as karaoke bars or Chinese restaurants tend to ignore such regulations. While having dinner or lunch, in moments of relaxation, they smoke a packet or more of tobacco. In their stores, Chinese individuals usually go outside to smoke since there are stricter regulations in these places. 

In the case of youth and women, cultural integration is more objective:

*“There is no difference in the tobacco and alcohol consumption among students and the new generation, but there is a difference in families between 30–40 years old”* (C-30, man, 41 years).

Awareness campaigns and health education in Spain, as well as the legal regulation of the sale of tobacco in specialized establishments and only to those over 18 years of age, have made access difficult for minors who have not become current smokers. However, smoking is considered not appropriate to women by the Chinese culture:

*“Women do not usually smoke because it is frowned upon”* (C-52, woman, 42 years); “The woman smoker is like … what it’s usually said here … a wh***” (C-43, woman, 36 years).

As it happens with tobacco, alcohol consumption has also a social acceptance within this community.

*“They drink a lot, it is essential in parties and celebrations (...). Chinese entrepreneurs eat at restaurants with other colleagues and order many expensive wines; they want the best to impress and treat business”* (C-10 man, 32 years).

There is a clear trend towards cultural integration when Chinese immigrants combine typical alcoholic beverages from both cultures and countries, such as beer and high-strength Chinese spirits.

*“Beer is becoming more popular and red wine is very common because this is the drink that best combines with Chinese food, but in family gatherings, more Chinese liquors are taken”* (C-131, woman, 27 years).

*“Chinese men drink a liquor called “bai jiu” (...). It’s a distilled 60-degree liquor. It is a national drink, like a brandy, which is taken with meals, to reach business agreements,* etc. *It’s a drink for Chinese [laughs]. We say „gānbēi” [cheers] and drink it in one shot”* (C-73 man, 34 years).

## 4. Discussion

Our results further advance the understanding of acculturation strategies among Chinese immigrants in Europe, particularly in Spain. According to our theoretical framework (Berry’s Model of Acculturation), our findings reveal that “separation” was still the predominant strategy, followed by “integration” and “assimilation”, while “marginalization” was not present in this Chinese immigrant population. In other words, most Chinese immigrants maintain a strong link to the customs of their home country, integrating few aspects of the host culture, which, in other words, favors the process of identity and collective self-esteem among this group.

This predominance of “separation” found in our sample has already been identified in a previous study, which included older Chinese immigrants in the United States. Considering that acculturation may influence eating patterns/diet, exercise, chronic disease, and mental health management, they found a strong identification of their participants with the Chinese culture (identificational acculturation), a high dependence on Chinese behavioral patterns and intraethnic networks, limited intergroup interactions (behavioral acculturation), and a strong maintenance of Chinese cultural values, while incorporating some American cultural learning (cognitive acculturation) [32]. The same patterns of “separation” were also observed in the Spanish context, in which Chinese immigrants tend to live in neighborhoods with a high density of Chinese individuals [33].

For Chinese immigrants, this cultural “separation” may be justified by the fact that, in the Chinese society, Confucianism posits the family as the fundamental unit of society, incorporating economic and social functions. Confucian values can be observed both in intergenerational relationships within the family and other social interpersonal relationships, which allow them to maintain the positive effect of family and community cohesion [34]. Current studies in Spain show that even younger Chinese immigrants of the second generation have a positive and strong sense of belonging towards their ethnic group. Nevertheless, they also believe that respect for the customs and traditions of both countries is important (“integration”), as well as good behaviors to be accepted by the new society [3]. In our case, since we investigated first-generation Chinese immigrants, this integration is less obvious and participants tended to maintain strong ties with their native culture. Nevertheless, it is important to note that younger Chinese immigrants tended to have a more open relationship with the host culture, as identified in the relationship with school friends and physical activity.

Even in older immigrants, the “integration” strategy can take place, as was revealed by the consumption of Western foods and the “forced” relationship with Spanish clients and co-workers. Health managers and health professionals should be aware of this aspect of the Chinese immigration in an attempt to value the Chinese culture, improving the adherence to treatment and interventions and also recognizing possible ways to integrate first-generation Chinese immigrants into the host culture. This integration is important since studies have demonstrated that biculturalism is strongly associated with better psychological and sociocultural adjustment, resulting in better health outcomes [35].

Despite the aforementioned strategies, our findings also revealed that prejudice exists among Spanish persons towards Chinese immigrants. According to Julián, high levels of prejudice on the part of the host population are linked to the preference for the attitude of assimilation, while low levels of prejudice are related to attitudes of integration [22]. According to previous studies in Europe, acquisition of nationality is an indicator of integration. This nationalization is usually motivated by an identity-related choice, but also as a utilitarian decision to deal with the economic crisis [36]. Different from other immigrants, Chinese individuals mostly choose to maintain their nationality, since they work in their own businesses and, for this reason, usually do not suffer unemployment or other economic problems that need nationalization [37]. This evident “separation” is noted by participants who reported avoiding prejudice in the relationship with Spanish individuals by using the tight support of the Chinese community and avoiding informal contact. 

Regarding health lifestyles, the acculturation process can be shown in different ways. On the one hand, if “separation” is common among older immigrants, than “integration” is more common among younger individuals. Corroborating our results, a Spanish study on eating habits of the Chinese immigrant population in Catalonia showed that the Chinese immigrants tried to maintain their diet of origin. On the other hand, the preference of the local diet by the children, work schedules, and lack of time were important barriers to keep this behavior [38]. A cross-sectional survey measured obesity risk reduction behavior and degree of acculturation among Chinese Americans. Asian-identified participants were most likely to follow traditional healthful Chinese food patterns, and Western-identified individuals were more apt to engage in leisure physical activity. Individuals categorized as bicultural were prone to use limited amounts of fats or oils when preparing foods [4]. These results reveal the positive effect on health derived from preserving at least part of the minority ethnic identity. Nevertheless, not all “integration” behaviors are harmful and the incorporation of sports for young immigrants proved to be beneficial to their health [39].

According to other authors, when the behavior is similar to those from the country of origin, it is possible that the behavior will be maintained by the migrant person and will be difficult to modified. For example, this situation can be better observed concerning the use of alcohol and tobacco [40], which was more prevalent in Chinese men in our study. On the other hand, immigrants could incorporate new behaviors to achieve integration, such as the increase in tobacco consumption in Chinese women [41].

Although some studies have concluded that Chinese migrants suffer a double marginalization and live in a constant balance between “out of place” and “in place” [42], marginalization (i.e., not identifying with any culture or having no interest in your own) was not a main issue for our participants.

In any case, ethnic minorities cannot always choose their preferred acculturation strategy, as the success of their acculturation strategy also depends on the larger society’s strategies (i.e., multiculturalism, melting pot, segregation, and exclusion) [12] and this should be considered while interpreting our findings.

These results have several clinical implications, revealing that Chinese immigrants still use “separation” as the predominant strategy, maintaining their cultural values and usually not incorporating aspects of the host’s culture. On the one hand, this approach helps them keep their values; on the other hand, it allows them to avoid integration with the host’s culture, which could in some occasions stigmatize them. Understanding the barriers to “integration” and valuing the native culture is essential to provide a more comprehensive and integrative treatment to these immigrants, improving their quality of life and health outcomes. Since acculturation is very distinct between younger and older individuals, as revealed in our study, intergenerational activities could be an important strategy to help in the “integration” process, since younger individuals tend to have a better relationship with the host community and can help decrease the “separation” and the fear of “assimilation” of their older counterparts.

Limitations and future directions for research are as follows: Owing to the lack of a standard methodology for measuring acculturation, acculturation orientations were used as a theoretical template to analyze ethnic minority’ discourses [32,43]. Although future studies could utilize bi/multidimensional scales and take into account the dynamic/transitory aspect of the acculturation process when studying health behaviors and still find discordant findings [31,44], the authors assume that the dynamic nature of the acculturation process can be lost when this theory is translated into a measurement instrument. In addition, new research should deepen investigations into the mixed vision of both an ethnic minority and an ethnic majority, since it allows for a better understating of acculturation outcomes [45]. Finally, this study was carried out before the COVID-19 pandemic. For this reason, different factors (e.g., migrants’ residency status, mobility, access to information, and healthcare) could have been modified int the immigrant population. Therefore, it is probable that health behaviors could have changed as well, modifying the patterns of acculturation previously studied [46,47].

## 5. Conclusions

The present study included the Chinese Migration Process using the Berry’s Model of Acculturation. The Chinese immigrant population shows a clear “cultural loyalty”, since the immigrants generally remain united with his/her compatriots, have little contact with the native population, and are interested in preserving the distinctive features of his culture of origin, resulting in the “separation” strategy. Although younger immigrants prefer to maintain their cultural heritage, they also seek contact with Spanish culture through the “integration” strategy.

According to our results, the most beneficial strategies for health are those where customs are maintained from the homeland, so “separation” and then “integration” are the most beneficial. Chinese cohesion offers social and economic support and protects them from perceived external threats. On the contrary, the strategy for assimilating Western Spanish customs can be a risk for their health status, highlighting the increased consumption of tobacco in Chinese women or the approach to a fatty nutritional pattern that could favor cardiovascular diseases. In this context, younger individuals seem to be more exposed to the “integration” strategy and could be important figures to reduce this “separation”.

Understanding of acculturation concept and its influence on health behaviors is helpful in identifying risk factors that underlie increased prevalence of chronic diseases and in designing intervention programs to reduce the burden of such diseases and to increase the quality of life in such populations. In addition, health interventions should be designed taking into account the dynamic nature of these acculturative responses and the differences that exist within the same ethnic group.

## Figures and Tables

**Table 1:** Development of the interview script.

Theoretical Framework	Authors Discussion	Themes	Questions
Reason for immigration, expectations for life in the new culture, role in the immigration decision, route and danger of migration, or time in the new culture are included in the framework of contextual factors influencing acculturation (Table S1).	There are different routes of entry for the immigrant population in Spain. There are groups of forced immigrants who come to Spain alone, while Chinese families are sometimes observed in their businesses. The migratory motive can generate different acculturation strategies according to the needs of each group (if they have more ethnic support or not).	Migratory process	What were the reasons for the migration? What has been your experience during migration and upon arrival in the host country? And over the years?
The acculturation model of Berry (1997) analyzes the results of contact between two cultures. In addition, separation from social support networks and loss of significant others are factors influencing acculturation (Table S1).	Social relation as a part of the contact between two culturally different groups. The presence of an ethnic support network can interfere with the way both cultures contact to each other. People are social beings, but do they need to feel part of the other culture or is ethnic support enough? Spanish people have prejudices towards the immigrant population, and those related to Chinese immigrants are of a legal or economic nature.	Social relations	What kind of relationships do you have with the Spanish population and your ethnic group?
According to the “Melting Pot” upon arrival in the host country, immigrants gradually take on cultural aspects and build a new “cultural form”. Immigrants usually change the way they dress, what they eat, their greeting procedures, and even their values by reducing their way of daily living, while taking on replacements.	What lifestyles are of concern in the world? And in Spain? We consider diet, physical activity, and substance use as the aspects that are the most explored and associated with chronic health problems and that represent a great expense for the national public health system.	Lifestyles	Since you are in Spain, what eating habits do you have? And what about physical activity? Have you started or changed alcohol or tobacco use?

**Table 2:** Sample characteristics.

Variables	Male	Female	Total	Statistics
	M (SD)	M (SD)	M (SD)	*p*-Value
Age (years)	33.1 (7.2)	29.2 (7.4)	30.7 (7.6)	U = 1457.5
				*p* =0.003
Years residing in Spain	12.7 (5.7)	10.4 (5.5)	11.3 (5.7)	U = 4774.5
				*p* = 0.017
	n (%)	n (%)	n (%)	
Sex	51 (38.3)	82 (61.7)	133 (100)	-
Marital status				
Single	11 (21.5)	34 (41.5)	45 (33.8)	χ^2^ = 7.35
Married	36 (70.6)	46 (56.1)	82 (61.7)	*p* = 0.062
Living with a partner (not married)	3 (5.9)	2 (2.4)	5 (3.8)	
Divorced	1 (2.0)	0 (0.0)	1 (0.8)	
Level of education				
Secondary or lower	41 (80.4)	62 (75.6)	103 (77.4)	χ^2^ = 0.41
Vocational or university	10 (19.6)	20 (24.4)	30 (22.6)	*p* = 0.521
Employment status				
Employed	50 (98.0)	78 (95.1)	128 (96.2)	χ^2^ = 0.74
Unemployed	1 (2.0)	4 (4.9)	5 (3.8)	*p* = 0.649

## Data Availability

The datasets used and/or analysed during the current study are available from the corresponding author on reasonable request.

## Supplementary Materials

The following are available online at https://www.mdpi.com/article/10.3390/ijerph18147639/s1, Table S1: Framework of contextual factors influencing acculturation; Table S2: Consolidated criteria for reporting qualitative studies (COREQ): 32-item checklist.
