# Supplementary material for: Acculturation, Health Behaviors, and Social Relations among Chinese Immigrants Living in Spain
Source: Int J Environ Res Public Health. 2021 Jul 18;18(14):7639. doi: 10.3390/ijerph18147639 (PMC8306044; doi:10.3390/ijerph18147639)
Supplement: Supplementary file 1 [file ijerph-18-07639-s001.zip › ijerph-1251231-supplementary.pdf]

Table S1: Framework of Contextual Factors Influencing Acculturation

**Prior immigration context**	
Society of origin factors	
Political, economic and social environment	Zhejiang is a rural geographical area, with few job and economic opportunities for its inhabitants, especially for those with a lower educational level.<br>China's notable economic growth in the past few decades has resulted in social economic inequality and, despite the fact that reforms to *hukou* (family registration system as a mechanism for controlling population movement) increased mobility among the population, both rural migrants and urban residents from other cities still face obstacles in accessing public benefits and services (Correa & Nuñez, 2013).
Individual factors	
Reason for immigration	Migration for economic reasons and then, for family grouping. The business success of the first generation of Chinese immigrants allowed the creation of an ethnic opportunity structure that is currently consolidated.
Role in the immigration decision	Chineses share this decision with other family members. One of the marriage partners emigrates alone and provides economic support to their family in China, waiting until the whole family is able to emigrate to Spain. The family agrees to help the person who emigrates, offering social and economic support.
Prior knowledge or contact with host society	None, only the experience of their peers in the host society.
Separation from social support networks and loss of significant others	Chineses maintain strong ties with the group of origin and search within their community for a support network that makes migration and adaptation to the destination a more comfortable and easier process. However, they miss their customs (totally different from Spanish customs), and the family they left in China, mainly parents and children.
**Immigration context**	
Type of immigration group	Family migration (nuclear type) of young people (30 years or less), with the man (husband) being the first to emigrate.
Route of immigration	Air route by plane. Chineses arrive in Spain from China to Barcelona, Madrid or Málaga. After that, expansion inwards (to other cities) according to places where their relatives or other compatriots live.
Level of danger in the immigration journey	Hazard not documented. Voluntary migration using flights (one of the safest routes).
Duration of immigration journey	20-30h (depending on the flight route, direct connection or not)
**Settlement context**	
Society of settlement factors	
Political (including immigration policies), economic and social environment	Organic Law 4/2000, of January 11, on Rights and Freedoms of Foreigners in Spain and their Social Integration. This law regulates the rights and obligations of foreigners in Spain and contains principles that seek to promote legal immigration, restricting to a minimum the entry of illegal foreigners and opportunities to normalize their situation for immigrants established in Spanish territory under conditions of irregularity. As a general rule, the Immigration Law establishes that it recognizes the rights established in the Spanish Constitution, international treaties, interpreted in

	accordance with the Universal Declaration of Human Rights, and other current treaties on citizen rights.
Societal attitudes toward immigrants	Some Chineses begin to interact with the Spanish population through work (e.g. customers, suppliers), but they mostly feel they receive normal or negative treatment, mainly related to cultural elements, stereotypes or different prejudices, such as feeling of guilt over unemployment.
Individual factors	
Age at time of settlement	Main family migrant: belonging to the working age group (30 years or less).<br>Other emigrants: children aged 5-6 and over
Legal and residency status	Most of the interviewees receive income from work (96.2%), and they have the assurance of having regular work. This implies a legal status.
Cultural distance between culture of origin and culture of settlement	Cultural differences in language (Spanish), religion (mainly Catholic), rituals and popular festivals, eating habits, conception of health-disease according to a Western medical approach.
Time in the new culture	The entry of Spain into the European Economic Community in 1986, made this country a good destination for the expansion of businesses by the Chinese population. Specifically, the sample of this study has an average length of residence in Spain of 11.3 years.
Expectations for life in the new culture	The expectations of the person who migrates is to be able to gather the rest of his family a few years later and continue the family business.<br>According to one participant and generally shared: "The idea is to work here and return to China (…) with a certain economic tranquility. But truly from the experience of my parents and their environment, in the end nobody has left".

Table S2: Consolidated criteria for reporting qualitative studies (COREQ): 32-item checklist.

No	Item	Guide questions/description	Response
Domain 1: Research team and reflexivity			
Personal Characteristics			
1.	Interviewer/facilitator	Which author/s conducted the interview or focus group?	All the interviews were conducted by the main author, BB.
2.	Credentials	What were the researcher's credentials? E.g. PhD, MD	B.B, R.d-D-C, M.G-C-C, M.G-C.M, J.V-E, A.B-R, S.B-T, D.d-P-J and GL was PhD. L.T.C was PhD student.
3.	Occupation	What was their occupation at the time of the study?	Researcher's occupations at the time of the study: student and research professor.

4.	Gender	Was the researcher male or female?	B.B, R.d-D-C, L.T-C, M.G-C-C, M.G-C.M were females. J.V-E, A.B-R, S.B-T, D.d-P-J and GL were males.
5.	Experience and training	What experience or training did the researcher have?	All researchers had experience in carrying out qualitative research. B.B and L.T.C has been trained to conduct interviews and R.d-D.C and M.G-C.M has training in social research.
Relationship with participants			
6.	Relationship established	Was a relationship established prior to study commencement?	No, there wasn't.
7.	Participant knowledge of the interviewer	What did the participants know about the researcher? e.g. personal goals, reasons for doing the research	Name, occupation, reasons for doing the research.
8.	Interviewer characteristics	What characteristics were reported about the interviewer/facilitator? e.g. Bias, assumptions, reasons and interests in the research topic	Name, occupation, contact method, reasons for doing the research.
Domain 2: Study design			
Theoretical framework			
9.	Methodological orientation and Theory	What methodological orientation was stated to underpin the study? e.g. grounded theory, discourse analysis, ethnography, phenomenology, content analysis	Phenomenological approach with a discourse and content analysis.
Participant selection			
10.	Sampling	How were participants selected? e.g. purposive, convenience, consecutive, snowball	Convenience sampling and snowball sampling.
11.	Method of approach	How were participants approached? e.g.	Face to face.

		face-to-face, telephone, mail, email	
12.	Sample size	How many participants were in the study?	133 Chinese immigrants and 7 key informants.
13.	Non-participation	How many people refused to participate or dropped out? Reasons?	279 for laboral reasons (mainly lack of time).
	Setting		
14.	Setting of data collection	Where was the data collected? e.g. home, clinic, workplace	Mainly in workplace, and other quiet and comfortable place chosen by the participant.
15.	Presence of non-participants	Was anyone else present besides the participants and researchers?	In all businesses there were other workers or family members (children among them).
16.	Description of sample	What are the important characteristics of the sample? e.g. demographic data, date	Chinese workers in their 30's emigrated to Spain.
	Data collection		
17.	Interview guide	Were questions, prompts, guides provided by the authors? Was it pilot tested?	Yes, they were. / Yes, it was.
18.	Repeat interviews	Were repeat inter views carried out? If yes, how many?	No, they weren't.
19.	Audio/visual recording	Did the research use audio or visual recording to collect the data?	Audio recording.
20.	Field notes	Were field notes made during and/or after the interview or focus group?	Yes, they were (field notes).
21.	Duration	What was the duration of the inter views or focus group?	Average 30-60 minutes (semi-structured interviws).
22.	Data saturation	Was data saturation discussed?	Yes, it was.
23.	Transcripts returned	Were transcripts returned to	Reviewed by 2 key informants.

		participants for comment and/or correction?	
Doman 3: Analysis and findings			
Data analysis			
24.	Number of data coders	How many data coders coded the data?	B.B, R.d-D-C, S.B-T
25.	Description of the coding tree	Did authors provide a description of the coding tree?	Yes, we did.
26.	Derivation of themes	Were themes identified in advance or derived from the data?	Themes were derived using both methods.
27.	Software	What software, if applicable, was used to manage the data?	NUDIST Nvivo 11.
28.	Participant checking	Did participants provide feedback on the findings?	Reviewed by 2 key informants.
Reporting			
29.	Quotations presented	Were participant quotations presented to illustrate the themes/findings? Was each quotation identified? e.g. participant number	Yes, there were. / Yes, there was.
30.	Data and findings consistent	Was there consistency between the data presented and the findings?	Yes, there was.
31.	Clarity of major themes	Were major themes clearly presented in the findings?	Yes, they were.
32.	Clarity of minor themes	Is there a description of diverse cases or discussion of minor themes?	Yes, there is.
